# The Digenic Causality in Familial Hypercholesterolemia: Revising the Genotype–Phenotype Correlations of the Disease

**DOI:** 10.3389/fgene.2020.572045

**Published:** 2021-01-15

**Authors:** Amina Kamar, Athar Khalil, Georges Nemer

**Affiliations:** ^1^Department of Biology, American University of Beirut, Beirut, Lebanon; ^2^Department of Biochemistry and Molecular Genetics, American University of Beirut, Beirut, Lebanon; ^3^Division of Genomics and Translational Biomedicine, College of Health and Life Sciences, Hamad Bin Khalifa University, Doha, Qatar

**Keywords:** digenic, familial hypercholesterolemia, *LDLR*, *PCSK9*, *APOB*, *LDLRAP1*

## Abstract

Genetically inherited defects in lipoprotein metabolism affect more than 10 million individuals around the globe with preponderance in some parts where consanguinity played a major role in establishing founder mutations. Mutations in four genes have been so far linked to the dominant and recessive form of the disease. Those players encode major proteins implicated in cholesterol regulation, namely, the low-density lipoprotein receptor (LDLR) and its associate protein 1 (LDLRAP1), the proprotein convertase substilin/kexin type 9 (PCSK9), and the apolipoprotein B (APOB). Single mutations or compound mutations in one of these genes are enough to account for a spectrum of mild to severe phenotypes. However, recently several reports have identified digenic mutations in familial cases that do not necessarily reflect a much severe phenotype. Yet, data in the literature supporting this notion are still lacking. Herein, we review all the reported cases of digenic mutations focusing on the biological impact of gene dosage and the potential protective effects of single-nucleotide polymorphisms linked to hypolipidemia. We also highlight the difficulty of establishing phenotype–genotype correlations in digenic familial hypercholesterolemia cases due to the complexity and heterogeneity of the phenotypes and the still faulty *in silico* pathogenicity scoring system. We finally emphasize the importance of having a whole exome/genome sequencing approach for all familial cases of familial hyperlipidemia to better understand the genetic and clinical course of the disease.

## Introduction

Familial hypercholesterolemia (FH) (MIM#143890) is a common inherited autosomal codominant disease with complete penetrance that is associated with high serum levels of low-density lipoprotein cholesterol (LDL-C) (Soutar and Naoumova, 2007; Castro-Orós et al., 2010; Singh and Bittner, 2015; Tada et al., 2019). The clinical diagnosis of FH is based on a family history of hypercholesterolemia (mainly in children), plasma LDL-C (>250 mg/dL or 7000 nmol/mL), and the presence of tendon xanthomas (Castro-Orós et al., 2010). Other external manifestations of the disease include xanthelasmas and corneal arcus, all of which are due to high LDL-C level (Khachadurian, 1988; Goldstein et al., 2001). The clinical distinction of FH was based on phenotypic severity of heterozygous and homozygous forms, where the LDL-C levels being two to four times, respectively, when compared to normal conditions (Khachadurian, 1964).

The prevalence of a heterozygous form of FH (HeFH) has a range of 1 in 250–500 people in most countries and is the most prominent genetic disorder leading to an increased risk of early onset of atherosclerotic cardiovascular disease (CVD) (Akioyamen et al., 2017; Pang et al., 2020). Atherosclerotic CVD is clinically manifested as coronary artery disease (CAD) and ischemic stroke, both of which are considered the leading causes of morbidity and death around the world (Ference et al., 2017; Moldovan and Banescu, 2020). However, the homozygous form of FH (HoFH) is rare and has a prevalence range of 1 in 160,000–1,000,000 worldwide (Brautbar et al., 2015; Singh and Bittner, 2015). The prevalence of HeFH has been reported to be higher in some populations with a pronounced founder effect due to high incidence of consanguineous marriages as is the case with the French Canadians, the South Africans, the Lebanese, and the Finns (Khachadurian, 1988; Koivisto et al., 1992; Goldstein et al., 2001). HeFH prevalence in South Africans and populations of some parts of Europe is 1:71 and 1:200, respectively (Steyn et al., 1989; Ito and Watts, 2015). In Lebanon, in contrast, a high reported frequency of HoFH was estimated to be approximately 1:85 in Christian Lebanese (Fahed et al., 2011; Brautbar et al., 2015). A less common autosomal recessive mode of inheritance has been detected in some of the initial families in Lebanon (Khachadurian, 1964).

For decades, familial autosomal dominant hypercholesterolemia (ADH) has been considered as a monogenic disorder in which more than 85% of the cases are mainly caused by mutations in *LDLR* (*low-density lipoprotein receptor*) (MIM#s 606945, 143890) (Pirillo et al., 2017; Tada et al., 2019; Alnouri et al., 2020). The *LDLR* gene is located on the short arm of chromosome 19 (19p13.1–p13.3) and spans around 45,000 base pairs (Brown and Goldstein, 1986). *LDLR* is made up of 18 exons, which encode a protein of 843 amino acids (Brown and Goldstein, 1986; Marais, 2004). More than 1,700 variants have been detected in the gene so far.^1^ Distinct mutations in other genes associated with LDL metabolism have also been shown to cause FH, including *APOB* (*apolipoprotein B*) (MIM #107730), *PCSK9* (*proprotein convertase substilin/kexin type 9*) (MIM # 607786), and *LDLRAP1* (*low-density lipoprotein receptor associate protein 1*) (MIM#605747) (Soutar and Naoumova, 2007; Singh and Bittner, 2015). *APOB* encodes two isoforms (apoB-48 and apoB-100) that constitute the protein component of the LDL-C particle and is considered the natural ligand of LDLR (Rader et al., 2003). PCSK9 encodes a convertase that is implicated in the internalization of the LDLR, whereas the *LDLRAP1* gene encodes a phosphotyrosine protein that directly interacts with the cytoplasmic tail of LDLR (Fahed and Nemer, 2011). Mutations in *LDLR* include deletions, insertions, point, and splicing mutations that disrupt the protein function (Wang et al., 2018). Those mutations lead to protein dysfunction through impairing LDL-C clearance from blood or depleting the number of LDLR within cells. Mutations in the *APOB* hinder the binding of apoB protein on the LDL particle with the LDLR and lead to familial defective APOB (FDB) or ADH2 (Damgaard et al., 2004; Vogt, 2015). Besides, a gain-of-function (GOF) mutation in *PCSK9* generates a hyperfunctioning PCSK9 protein, which in turn increases LDLR degradation and causes ADH3 (Vogt, 2015). Conversely, loss-of-function (LOF) mutations lead to an increase in LDLR expression on the cells and diminished LDL-C levels (Nordestgaard et al., 2013; Cohen et al., 2006). Therefore, the incidence of CVD is significantly reduced in individuals with LOF mutations and lower LDL-C levels, which supports the suggestion that low levels of LDL-C from the time of birth are associated with lower cardiovascular risk (Cohen et al., 2006).

Concomitant with the discovery of the additional genes, high-throughput sequencing analysis has revealed a more complicated “polygenic” form of FH with varying degrees of severity, which was further enhanced by the discovery of modifier genes (Fahed et al., 2016; Tada et al., 2019). The latter are defined as genes involved in altering the course of disease where their protein products become immediate targets for therapeutic intervention (Cutting, 2010). While monogenic FH is predominantly caused by a mutation in a single gene, polygenic FH is usually caused by alterations in two genes or more (Narayanaswamy and Sharma, 2020). Oligenic FH has also been introduced and indicates the presence of damaging mutations in the standard FH genes and LDL-altering accessory genes (Tada et al., 2018). The latter comprises multiple low-frequent variants of genes associated with lipid-related autosomal diseases, including LDLRAP1, adenosine triphosphate–binding cassette subfamily G, member 5/8 (ABCG5/8), and apolipoprotein E attributed to a more severe FH phenotype (Tada et al., 2019). Another severe autosomal recessive FH (ARH, MIM ID #603813) is caused by an LOF mutation in *LDLRAP1* (Soutar and Naoumova, 2007). Comparable to the *LDLR* case, some *LDLRAP1* mutations are geographically associated with a noticeable founder effect such as in Europe and Lebanon (Garcia et al., 2001; Wilund et al., 2002; Cohen et al., 2003; Pisciotta et al., 2006a; Quagliarini et al., 2007; Fahed et al., 2016).

Mutations identified in the primary FH-causing genes (including *LDLR*, *APOB*, *PCSK9*, and *LDLRAP1*) show a similar gene dosage effect and share a comparable clinical phenotype, describing a semidominant inheritance pattern (Nordestgaard et al., 2013). Although mutations in these genes have comparable phenotypes, these still vary in severity and LDL-C blood levels (Brautbar et al., 2015). However, the number of mutant alleles does not always reflect the severity of the disease phenotype, and this was revealed by the variable expressivity of mutations in FH (Fahed and Nemer, 2011; Fahed et al., 2016). Yet, the considerable proportion of adults with FH (∼17–33%) without any detectable variation in the four mentioned genes points out to the likelihood of other unknown genes involved in the pathogenesis of FH (Castro-Orós et al., 2010; Paththinige et al., 2017). For this reason, recent studies are suggesting a combinatorial effect of single mutations that can affect various genes in the LDLR pathway and can yield a phenotype intermediate between heterozygous and homozygous. This, in return, reveals that the genetic heterogeneity of FH among various populations can aid in unraveling other unknown genes that might be involved in FH pathogenesis (Wang et al., 2011). However, the variability in expression creates a gap and makes it challenging to establish phenotype–genotype correlations in FH cases. Genotype–phenotype correlations in FH are so far essential as they permit a better clinical evaluation of the severity of phenotype generated by the mutation and its treatment response (Fahed et al., 2011). Usually, physicians use “homozygous” and “heterozygous” terms to designate the phenotype of FH patients. In this case, HoFH and HeFH should refer only to LDL levels and not to the genotype (Fahed and Nemer, 2011). This could be explained by the fact that some heterozygous mutations in FH patients could be very severe, with LDL-C levels being four times more than normal conditions, thus falling under HoFH. At the same time, the opposite could also occur (Fahed et al., 2016). Therefore, the clinical nomenclature of FH was classified into three categories, including (1) severe, (2) mild, and (3) paradoxical. The latter reveals cases with a confusing presentation of FH. Most FH patients receive high-dose statin therapy. However, many of these patients are still unable to achieve the desired lipid levels and therefore require additional treatments, including LDL apheresis (Moyer and Baudhuin, 2015).

Although the clinical features of both heterozygous and homozygous mutations in monogenic FH have significantly been described, very few is known about “double-heterozygous” or digenic FH phenotypes, where we have a combination of mutations between any two of the known FH-causing genes including *APOB, LDLR, PCSK9*, and *LDLRAP1* (Tada et al., 2011; Cuchel et al., 2014). The Consensus Panel on Familial Hypercholesterolemia of the European Atherosclerosis Society has previously stated that the general levels of LDL-C in double-heterozygous carriers of mutations in *APOB*, *PCSK9*, or *LDLR* to be less severely elevated as compared to homozygous carriers of variations in the same genes (Cuchel et al., 2014). However, data in literature supporting this statement are still lacking (Sjouke et al., 2016b). Therefore, we will review all the reported digenic (double-heterozygotes) cases of FH with *LDLR*, *PCSK9*, *APOB*, and *LDLRAP1* mutations. We will also dissect the pathogenicity of the phenotypes and the molecular effect of the digenic interactions if present, in addition to the efficacy of the proposed therapies on improving the phenotypes. Finally, we will highlight the importance of next-generation sequencing (NGS) testing for all FH cases in unraveling new genes involved in this heterogeneous disease.

## Digenic Familial Hypercholesterolemia Mutations

Digenic inheritance (DI), previously termed epistasis, is the simplest form of oligogenic inheritance for genetically complex diseases (Schäffer, 2013; Gazzo et al., 2016). In oligenic FH, multiple rare mutations contribute to a more severe phenotype (Tada et al., 2019). More recently, Schäffer (2013) has preferably introduced DI as when variant genotypes at two genes explain the phenotypes of some patients and their mildly affected or unaffected relatives more clearly than genotypes in one gene, reflecting a reduced penetrance of the disorder. Rarely, in this case, the patients coinherit two separate genetic mutations, also described as “double” or transheterozygosity (Deltas, 2018). The combinatorial interaction of mutations on two genes is needed in this case to cause the disease (Vockley, 2011). There are very few diseases with a digenic mode of inheritance, and FH has shown to be one of them (Deltas, 2018).

Since the discovery of the *LDLR* gene by Brown and Goldstein (1986), numerous pathogenic mutations, including those in the *APOB* and *PCSK9* genes, have been identified along with the advancement and standardization of genetic analysis (Mabuchi, 2017). In monogenic FH, pathogenic mutations in one gene may be responsible for the expression of the disease phenotype, which also explains a defined diagnosis (Deltas, 2018). The known causative mutations in the *LDLR* gene account for the majority of FH cases with a range of 90–95%, along with 5–10% for *APOB*, and a maximum of 3% for *PCSK9* (Benn et al., 2016). Moreover, pathogenic mutations in the *LDLRAP1* gene are infrequent (up to two variants) in patients with FH (Spina et al., 2018). However, FH patients, in extreme cases, might have *LDLR* mutations with LDL-C levels falling within the normal range, which leads to reduced penetrance. The variability in expression of monogenic FH patients carrying the same gene mutation suggests other genetic or environmental interactions (Benlian et al., 1996).

**FIGURE 1:**
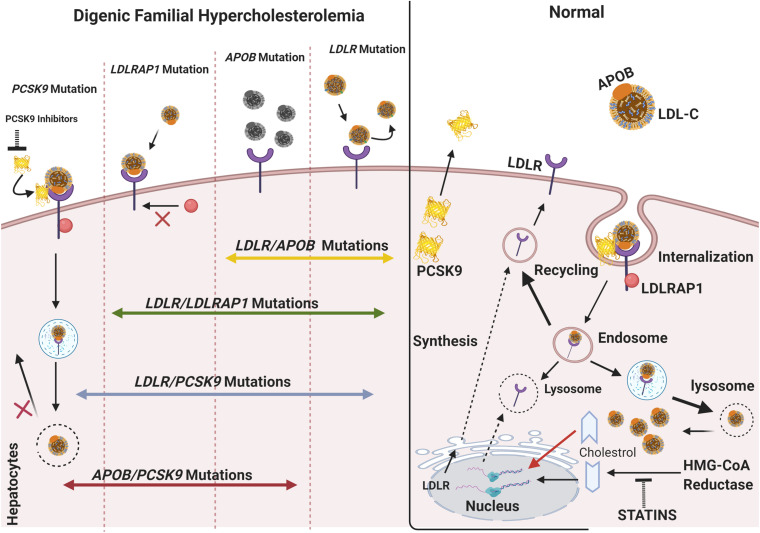
The molecular basis of digenic familial hypercholesterolemia. In normal cases, the LDLR located on the surface of hepatocytes binds APOB/LDL-C forming a complex, which is endocytosed into the cell via the interaction of LDLR with LDLRAP1. As the complex dissociates inside the cell, LDLR usually recycles to the cytoplasmic membrane, and the free cholesterol is consumed inside the cell. Posttranscriptional inhibition of LDLR is mediated by PCSK9 through cell-surface interactions. The transcription of *LDLR* is activated in response to the decrease in cholesterol levels in the cell, such as during treatment with statins. However, an increase in the level of intracellular cholesterol induces ubiquitin-mediated LDLR degradation. In monogenic FH, single mutations in *LDLR*, *APOB*, *PCSK9*, and *LDLRAP1* account for a broad spectrum of phenotypes ranging from mild to severe. *LDLR* mutations affect the binding of LDL-C to LDLR and cause the accumulation of LDL-C in circulation. *APOB* mutations cause protein dysfunction and lead to a limited binding of LDL-C to LDLR. Mutations in *PCSK9*, usually GOF, can account for (1) an increased PCSK9 activity, (2) no LDLR recycling, and (3) accumulation in plasma LDL-C levels. In such a case, anti-PCSK9 therapies, including the emerging PCSK9 inhibitors (e.g., alirocumab, evolocumab), are used to lower LDL-C and prevent CVD. In digenic familial hypercholesterolemia, double-heterozygotes can include an *LDLR* mutation inherited from one parent and a second mutation in *APOB*, *LDLRAP1*, or *PCSK9* inherited from either the same or the other parent. A mutation in *PCSK9* can also occur with another *APOB* mutation. The combination of two mutations in the main FH-causing gene does not account for a more severe phenotype all the time. The phenotype can thus be similar to that of monogenic FH and can cause an impaired LDL-C clearance. The figure was created using https://biorender.com/.

In some cases reported, FH patients displayed digenic mutations with two different heterozygous mutations in two loci at separate genes, including the involvement of *LDLRAP*, *APOB*, or *PCKS9* (Moyer and Baudhuin, 2015; Figure 1). Below, we describe all the FH cases reported in the literature with digenic mutations with an emphasis on the phenotype on one hand and the pathogenicity of mutations on the other hand. The outreaching goal is to highlight the minimal significance of the identified genetic variants in explaining the heterogeneity of the disease even when combining the variants together.

According to our findings, *LDLR/APOB* mutations are the most common among these digenic mutations in FH. While *APOB/PCSK9* mutations are still available, they are less common. Despite being rare, the cases mentioned below provide insight into the molecular mechanisms that underlie the clinical and phenotypic variability in cases with digenic mutations in the primary FH-causing genes (Table 1).

**TABLE 1:** A summary of double-heterozygote FH cases and their characteristics as reported in literature.

**Digenic Mutation/Number of Individuals Identified (N)**	**Nomenclature of Variants identified**	**Gender**	**Age, years**	**Untreated LDL-C (nmol/mL)**	**Phenotype severity**	**Treatment**	**Response to treatment (%LDL-C reduction)**	**References**
***APOB/LDLR*** (N=1)	p.Arg3500Gln+LDLR defect (NA)	M	35	7010	Severe (similar to FH and FDB)	NR	NR	Rauh et al., 1991
***APOB/LDLR*** (N=7)	p.Arg3500Gln+p.Asp206Glu	F	48	13600	Intermediate severity with no CAD	Simvastatin 20	25.4%	Rubinsztein et al., 1993; Raal et al., 1997
	p.Arg3500Gln+p.Asp206Glu	F	26	13800	Intermediate severity with no CAD	Simvastatin 20	25.4%	
	p.Arg3500Gln+p.Asp206Glu	F	21	100000	Intermediate severity with no CAD	Simvastatin 20	25.4%	
	p.Arg3500Gln+p.Asp206Glu	F	43	14680	Intermediate severity with no CAD	Simvastatin 20	25.4%	
	p.Arg3500Gln+p.Asp206Glu	M	23	9200	Intermediate severity with no CAD	Simvastatin 20	25.4%	
	p.Arg3500Gln+p.Asp206Glu	F	2	7900	Intermediate severity with no CAD	NR	NR	Rubinsztein et al., 1993
	p.Arg3500Gln+p.Asp206Glu +Val408Met)	M	12	20790	Very Severe with premature atherosclerosis	Partial Ileal bypass surgery	35% reduction in Total cholesterol, Asymptomatic Reduction in LDL-C NR	
***APOB/LDLR*** (N=2)	p.Arg3500Gln +p. Trp66Gly	M	10	15070	Severe	simvastatin 20, cholestyramine 16g	46%	Benlian et al., 1996
	p.Arg3500Gln+p.Glu207Lys	M	55	10630	Severe	simvastatin 40 + cholestyramine 24g	48%	
***APOB/LDLR*** (N=1)	p. Arg3527Gln+p. Gly20Arg	M	48	6260	Mildly severe	10 mg/dl of atorvastatin	43%	DeCampo et al., 2001
***APOB/LDLR*** (N=1)	p.Arg3500Trp+p.Asp407Lys	F	43	9470	Very severe (Exaggerated FH)	Simvastatin 80	49%	Tai et al., 2001
***APOB/LDLR*** (N=1)	p.Arg3527Gln+p.Leu479Pro	F	15	8800	Severe with premature CAD	Atorvastatin 20	34%	Taylor et al., 2010
***APOB/LDLR*** (N=2)	p.Arg3558Cys+p.Tyr19*	M	43	6900	Xanthomas (LDL-c level Similar to LDLR heterozygotes)	NR	NR	Bertolini et al., 2013
	p.Arg3558Cys+p.Tyr19*	M	12	7340	LDL-C level similar to LDLR heterozygotes			
***APOB/LDLR*** (N=23)	p.Arg3527Gln+c.31311G>A	M	73	NR	Intermediate Severe	Rosuvastatin 20, Ezetimibe 10	25.4% reduction reported in 5 patients	Sjouke et al., 2016a
	p.Arg3527Gln+c.31311G>A	F	46	NR	Intermediate Severe	Atorvastatin 80, Ezetimibe 10		
	p.Ser4430Thr+p.Trp87Arg	M	37	6000	Intermediate Severe	NR		
	p.Arg3558Cys+p.Trp44*	F	46	NR	Intermediate Severe	Fluvastatin 40, Ezetimibe 10		
	p.Arg3527Gln+ p.Glu228Lys	M	16	8800	Intermediate Severe	Pravastatin 30, Ezetimibe 10		
	p.Arg3527Gln+p.Gly98Ser	M	51	6200	Intermediate Severe	Rosuvastatin 40, Ezetimibe		
	p.Arg3527Gln+p.Gly98Ser	M	24	6800	Intermediate Severe	Rosuvastatin 20		
	p.Arg3527Gln+ p.Asp535Asn	F	61	8200	Intermediate Severe	Rosuvastatin 10		
	p.Arg3527Gln +p.Asp535Asn	F	58	NA	Intermediate Severe	Rosuvastatin 10		
	p.Arg3527Gln+p.Asp535Asn	F	31	7400	Intermediate Severe	Atorvastatin 20		
	p.Arg3500Trp +p.Cys184Arg	F	69	7100	Intermediate Severe	None		
	p.Arg3500Trp+p.Cys184Arg	F	42	13100	Intermediate Severe	Rosuvastatin 40, Ezetimibe 10		
	p.Arg3527Gln+ p.Asn564His/p.Val800_ Leu802del	M	64	NA	Intermediate Severe	Rosuvastatin 40, Ezetimibe 10		
	p.Arg3527Gln +p.Asn564His/p.Val800_ Leu802del	F	62	9100	Intermediate Severe	Rosuvastatin 40, Ezetimibe 10		
	p.Arg3527Gln +p.Asn564His/p.Val800_ Leu802del	M	55	NR	Intermediate Severe	Simvastatin 20, Ezetimibe 10		
	p.Arg3527Gln+p.Asn564His/p.Val800_ Leu802del	F	36	8700	Intermediate Severe	Rosuvastatin 40		
	p.Arg3527Gln+p.Asn564His/p.Val800_ Leu802del	M	33	NR	Intermediate Severe	Atorvastatin 40, Ezetimibe 10		
	p.Arg3527Gln +c.31311G > A	F	25	6200	Intermediate Severe	Atorvastatin 40		
	p.Phe807Leufs*10+ p.Arg3527Gln	M	6	7400	Intermediate Severe	Pravastatin 40		
	p.Arg3527Gln +p.Ala705Pro	F	32	9600	Intermediate Severe	Atorvastatin 80, Ezetimibe 10		
	p.Arg3527Gln+p.Ser306Leu	M	70	NR	Intermediate Severe	Rosuvastatin 10, Ezetimibe 10, Cholestagel 2500, Gemfibrozil 900		
	p.Gln4376*+c.19014A >T	F	53	NR	Intermediate Severe	Atorvastatin 80, Ezetimibe 10		
	p.Arg3558Cys+p.Cys255Arg	F	57	NR	Intermediate Severe	Atorvastatin 40		
***APOB/LDLR*** (N=5)	p.Arg3527Trp+p.Trp490Cys	NR	NR	NR	NR	NR	NR	Pirillo et al., 2017
	p.Arg3527Trp+p.Asp579Asn	NR	NR	NR	NR	NR	NR	
	p.Arg3527Trp+ p.Asp221Gly	NR	NR	NR	NR	NR	NR	
	p.Arg3527Trp+p.Asp221Gly	NR	NR	NR	NR	NR	NR	
	p.Arg3527Trp+p.Asp221Gly	NR	NR	NR	NR	NR	NR	
***APOB/LDLR*** (N=6)	p.Arg3527Gln+c.1846-?_21401?del	F	59	4315	High CVD risk	Rosuvastatin 20, Ezetimibe, Alirocumab 75/150 mg Q2W	39.3% to 55.7%	Hartgers et al., 2018
	p.Arg3527Gln+.2390-?_25831?del	F	50	4008	High CVD risk	Rosuvastatin 40, Alirocumab 150 mg Q2W	39.3% to 55.7%	
	Arg3527Gln+p.Asp227Glu	F	69	7500	High CVD risk	Atorvastatin 80, Ezetimibe, Alirocumab 75/150 mg Q2W	55.1% to 62.0%	
	p.Arg3527Gln+p.Cys209Tyr	F	47	3879	High CVD risk	Rosuvastatin 40, Ezetimibe, Alirocumab 75/150 mg Q2W	55.1% to 62.0%	
	p.Arg3527Gln+p.Tyr375Trpfs*7	F	58	6956	High CVD risk	Atorvastatin 80 mg Ezetimibe, fish oil, Placebo	NR	
	p.Arg3527Gln+p.Gly478Arg	M	54	4215	High CVD risk	Atorvastatin 80, Ezetimibe, nicotinic acid, Placebo	NR	
***APOB/PCSK9*** (N=1)	p. Ala3396Thr +p. Arg96Cys	M	NR	11378	Severe with a history of MI	Statin treatment	74%	Elbitar et al., 2018
***APOB/LDLR* or *LDLR/PCSK9*** (N=13)	NR	NR	46 ± 12	6890 ± 1120	Mild, 4 patients with CAD family history	8 treated with Statins	NR	Sun et al., 2018
***LDLR/PSKC9*** (N=3)	p.Glu228Lys+p.Arg496Trp	F	35	1152	Severe (similar to homozygous FH), Xanthomas and CHD	Atorvastatin 40 alone or more recently in combination with ezetimibe 10, and selective LDL-apheresis (twice a month)	∼ 65–70%	Pisciotta et al., 2006a; Bertolini et al., 2013
	p.Tyr419*+p.Asn425Ser	F	48	13310	Severe (similar to homozygous FH), xanthomas	Simvastatin 80, cholestyramine (24 g/day)	40%	
	p.Tyr419*+p.Asn425Ser	F	62	10470	Severe (similar to homozygous FH)		40%	
***LDLR/PCSK9*** (N=5)	c.191-2A.G+ p.Arg476Cys	M	51	10300	Intermediate severe (as compared to heterozygous, homozygous and compound heterozygotes)	Simvastatin 80, Ezetimibe 10	40.7%	Sjouke et al., 2016a
	c.1912A.G+p.Arg476Cys	M	79	NR	Intermediate severe (as compared to heterozygous, homozygous and compound heterozygotes)	NR	NR	
	c.191-2A.G+p.Arg476Cys	M	83	NR	Intermediate severe (as compared to heterozygous, homozygous and compound heterozygotes)	Atorvastatin 40, Ezetimibe 10	NR (LDL-C treated= 3200 nmol/mL)	
	p.Val429Met+p.Ala53Gly	F	64	NR	Intermediate severe (as compared to heterozygous, homozygous and compound heterozygotes)	Atorvastatin 80, Ezetimibe 10, cholestagel 2500	NR (LDL-C=3600nmol/mL)	
	p.Val429Met+ p.Ala53Gly	M	60	14800	Intermediate severe (as compared to heterozygous, homozygous and compound heterozygotes)	Rosuvastatin 40, Ezetimibe 10	76.35%	
***LDLR/PCSK9*** (N=17)	c.1187-?_2140þ?dup p.(Gly396_Thr713dup)+c.1327G>A p.(Ala443Thr)	NR	NR	NR	NR	NR	NR	Pirillo et al., 2017
	c.1246C>T (2 subjects) p.(Arg416Trp) +C.137G>T p.(Arg46Leu) LOF variant	NR	NR	NR	NR	NR	NR	
	c.1257C>G p.(Try419*) c.60_65dupGCTGCT (c.61_63triCTG) p.(Leu21tri)	NR	NR	NR	NR	NR	NR	
	c.1257C>G p.(Try419*) C.137G>T p.(Arg46Leu)	NR	NR	NR	NR	NR	NR	
	c.126C>A p.(Try42*) C.137G>T p.(Arg46Leu)	NR	NR	NR	NR	NR	NR	
	c.1646G>A p.(Gly549Asp) c.60_65dupGCTGCT (c.61_63triCTG) p.(Leu21tri)	NR	NR	NR	NR	NR	NR	
	c.1783C>T p.(Arg595Trp) c.60_65dupGCTGCT (c.61_63triCTG) p.(Leu21tri)	NR	NR	NR	NR	NR	NR	
	c.1846-?_2583þ?del (2 subjects) p.0 Pathogenic C.137G>T p.(Arg46Leu)	NR	NR	NR	NR	NR	NR	
	c.2215C>T p.(Gln739*) C.137G>T p.(Arg46Leu)	NR	NR	NR	NR	NR	NR	
	c.2312-3C>A p.(Ala771_Ile796del) C.137G>T p.(Arg46Leu)	NR	NR	NR	NR	NR	NR	
	c.352G>T p.(Asp118Tyr) C.137G>T p.(Arg46Leu)	NR	NR	NR	NR	NR	NR	
	c.373C>T (HO) p.(Gln125*) c.60_65dupGCTGCT (c.61_63triCTG) p.(Leu21tri)	NR	NR	NR	NR	NR	NR	
	c.418G>T p.(Glu140*) C.137G>T p.(Arg46Leu)	NR	NR	NR	NR	NR	NR	
	c.418G>T p.(Glu140*) c.60_65dupGCTGCT (c.61_63triCTG) p.(Leu21tri)	NR	NR	NR	NR	NR	NR	
	c.662A>G p.(Asp221Gly) C.137G>T p.(Arg46Leu)	NR	NR	NR	NR	NR	NR	
	c.788A>G p.(Asp263Gly) NEW c.-331C>A	NR	NR	NR	NR	NR	NR	
	c.1547G>A p.(Gly516Asp) c.60_65dupGCTGCT (c.61_63triCTG) p.(Leu21tri)	NR	NR	NR	NR	NR	NR	
***LDLR/PCSK9*** (N=1)	p.Cys143+p.Leu22_ Leu23dup	M	54	3157	NR	Rosuvastatin 40, ezetimibe, alirocumab 150, Q4w	NR	Hartgers et al., 2018
***LDLR/PCSK9*** (N=1)	p.I531TfsX15+ V4I	M	38	7030	Severe with ST MI, Achilles tendon Xanthoma, prone to CAD	Rosuvastatin 10, Ezetimibe 10, Evolocumab 140 mg administred every two weeks	∼ 85.2 %	Shirahama et al., 2018
***LDLR/LDLRAP1*** (N=4)	p.Q233P+p.Q136X	M	28	14680	Severe	Atorvastatin 80, 12 g cholestyramine 10, Ezetimibe, and LDL Aspheresis	Target was not reached by drugs, Therefore, they did aspheresis	Soufi et al., 2013
	p.Q233P+p.Q136X	M	2	12920	Severe			
	p.Q233P+p.Q136X	M	1	9150	Severe			
	p.Q233P+p.Q136X	F	24	5570	Severe			
***LDLR/LDLRAP1*** (N=3)	(c.2431A>T)+(c.606dup)	F	79	8223	Severe, xanthomas, Coronary and aortic vulvar Disease	Statin Therapy, suspended during this study	NR	Tada et al., 2011
	(c.2431A>T)+(c.606dup)	M	45	60700		Statin Therapy, suspended during this study	NR	
	(c.2431A>T)/(c.606dup)	F	32	7732		Statin Therapy, suspended during this study	NR	
***LDLR/LDLRAP1*** (N=4)	c.1255 T > G, p.(Y419D)+ [c.604_605delTCinsA, p.(S202Tfs*2)].	F	45	12490	Severe, STEMI, xanthomas, CAD family history	Rosuvastatin, Ezetimibe	Lowered, but not as recommended by ESC	Alnouri et al., 2018
	c.1255 T > G, p.(Y419D)+ [c.604_605delTCinsA, p.(S202Tfs*2)].	M	17	5500	Severe, CAD family history	None	None	
	c.1255 T > G, p.(Y419D)+ [c.604_605delTCinsA, p.(S202Tfs*2)].	M	14	NA	Severe, CAD family history	None	None	
	c.1255 T > G, p.(Y419D)+ [c.604_605delTCinsA, p.(S202Tfs*2)].	F	8	5.3	Severe, CAD family history	None	None	
***LDLR/LDLRAP1*** (N=9)	*LDLR* hetero C681+*LDLRAP* hetero Q136* (N=2)	NR	NR	∼6150	No universal pattern identified, Xanthomas (N=1)	NR	NR	Fahed et al., 2016
	*LDLR* homo C681*+*LDLRAP* hetero Q136* (N=1)	NR	NR	12852	No universal pattern identified Xanthomas, Xanthelasmas	NR	NR	
	*LDLR* hetero A391T + *LDLRAP* hetero Q136* (N=4)	NR	NR	4500	No universal pattern identified	NR	NR	
	*LDLR* hetero A391T + *LDLRAP* homo Q136* (N=1)	NR	NR	15050	No universal pattern identified	LDL Aspheresis	NR	
	*LDLR* hetero C681* + *LDLR* hetero A391T + LDLRAP hetero Q136 (N=1)	NR	NR	7861	No universal pattern identified	NR	NR	
***LDLR/LDLRAP1*** (N=1)	p.(Glu228Lys)+ p.(Glu250Glyfs*4)	NR	NR	NR	NR	NR	NR	Pirillo et al., 2017

*NR: not reported; Drug doses are in mg/day; CVD: Cardiovascular disease; Q2W, every 2 weeks; Q4W, every 4 weeks, MI: Myocardial Infarction, HoFH: homozygous FH; ESC: European Society of Cardiology; STEMI: ST-elevation myocardial infarction.*

### *LDLR/APOB* Mutations

Several genetic defects in the *LDLR* gene, which encodes the LDL receptor (LDLR) is the main cause of FH (ADH1). Generally, FH patients with mutations in *LDLR* reveal the most severe phenotypes, including high LDL-C level and a low response to lipid-lowering therapies (LLT) (Fahed and Nemer, 2011). However, *APOB* mutations have incomplete penetrance, and patients with FDB have less severe phenotypes when compared to FH patients having *LDLR* variants. As the LDL-C uptake is lowered upon *LDLR* mutations, a similar pattern is sometimes obtained if there is a mutation in *APOB* (Soufi et al., 2004). For FDB, the p.(R3500Q) variant is the most prevalent, and the first to be identified. The p.(R3500Q) is common in Europe and accounts for 2–5% of FH phenotypes (Myant, 1993). Other point mutations in the *APOB*, including p.(R3500W) and p.(R3531C), are rarely occurring, all of which are responsible for impairing the binding of LDL-C to LDLR (Soria et al., 1989; Myant, 1993; Gaffney et al., 1995; Pullinger et al., 1995). FDB patients are assumed to exhibit the same clinical features as FH caused by a defective LDLR (Rauh et al., 1991).

The Ldlr^–/–^ FH mice model revealed a marked elevation in LDL-C and apoB levels and developed acute atherosclerosis upon a chow diet (Powell-Braxton et al., 1998). Patients with compound *LDRL* (ADH) and *APOB* (FDB) mutations are commonly rare, with a prevalence of 1 in 564 (∼0.185), where very few studies have described their occurrence (Myant, 1993; Pirillo et al., 2017). Initially, a study done in 1991 reported an FH double-heterozygous (termed “heterozygous compound”) individual with a p.(Arg3500Gln) paternal *APOB* mutation along with a non-reported defective maternal *LDLR*. Unexpectedly, the clinical features of the individual with double mutations were similar to those of family members with either defect, including tendon xanthoma and premature atherosclerosis (Rauh et al., 1991). One explanation for this was that the degree of expression of the mutated *LDLR* and *APOB* in the double-heterozygous individual is different than that of the family members. The prognosis and treatment of this individual were recommended to be different, suggesting that other genes or environmental factors might be affecting this phenotype (Rauh et al., 1991). Similarly, Rubinsztein et al. (1993) reported a South African family who had five double-heterozygous individuals carrying the p.(Asp227Glu) *LDLR* and p.(Arg3527Gln) *APOB* mutations, and one “complex” heterozygote who also accounted for a p.(Val429Met) *LDLR* mutation. The double-heterozygous carriers had lipid levels and clinical features that were intermediate in severity between heterozygous and homozygous FH. The daily treatment of these individuals with Simvastatin 20 mg for 6 weeks reduced the LDL-C level by 25.4% (Raal et al., 1997). Also, Benlian et al. (1996) reported a double-heterozygote mutation in *LDLR* and *APOB* in two unrelated French patients with no family history for atherosclerosis. These patients were described as a “new class” of patients with digenic lipid disorders, defined by specific clinical features that result from the combined effects of two independent loci. The two patients were double-heterozygous for the p.(Arg3500Gln) *APOB* mutation and either the p.(Trp66Gly) or p.(Glu207Lys) *LDLR* mutations reflecting an unusual phenotype of “aggravated hypercholesterolemia.” The two missense mutations in LDLR were previously detected in a French Canadian cohort, and the p.(Arg3500Gln) *APOB* is a founder mutation in Northern Europe (Leitersdorf et al., 1990; Benlian et al., 1996). Interestingly, their phenotypes were different than those of their heterozygous FH relatives and the homozygous FH and FDB patients. The reported phenotype was also complicated with premature CAD, although remaining responsive to lipid-lowering drugs. The latter included daily uptake of simvastatin 20 mg along with 16 g of cholestyramine. This treatment managed a long-term decrease in the level of plasma lipids (Benlian et al., 1996). The observed phenotype of aggravated hypercholesterolemia gives further evidence that the *LDLR* and *APOB* play distinct roles in regulating the metabolism of LDL. Although both phenotypes were severe, one case expressed a milder phenotype compared with the other, which represented a disease closer to that reported in FH homozygotes carrying the p.(Trp66Gly) mutation (Moorjani et al., 1993). One explanation for this is that the clinical variability of FH depends on the type of mutation (Hobbs et al., 1992; Kotze et al., 1993). It was reported that p.(Glu207Lys) *LDLR* mutation reduces the rate of transport of native LDLR receptors to the surface of the cell, while the p.(Trp66Gly) *LDLR* mutation causes severe functional defects *in vitro* (Leitersdorf et al., 1990; Hobbs et al., 1992). Another explanation for this phenotype is that the abnormal presence of defective LDLR *in vivo* affected the expression of the wild-type allele in FH heterozygotes carrying the p.(Trp66Gly) *LDLR* mutation (Moorjani et al., 1993). At the molecular level, these patients suggested to produce a functional *LDLR* and an apoB ligand. However, they should have a particular therapeutic intervention (Benlian et al., 1996).

However, DeCampo et al. (2001) reported a double-heterozygous case carrying the p.(Arg3500Gln) *APOB* and p.(Gly2Arg) *LDLR* mutations. Yet, his plasma LDL-C level was intermediate when compared to other family members with biallelic mutations. The daily treatment with 10 mg/dL of atorvastatin returned his plasma LDL-C to normal (DeCampo et al., 2001). Another study done by Tai et al. (2001) reported a double-heterozygous case from an Asian family carrying the p.(Asn428Lys) *LDLR* and p.(Arg3527Trp) *APOB* mutations. The detected proband had a pretreatment serum LDL-C level approximately twice as high as her siblings. The reduction in LDL-C level after the treatment was similar to the two cases reported by Benlian et al. (1996) and Tai et al. (2001) before. The p.(Asp407Lys) mutation in the *LDLR* gene of this patient falls within exon 9. It thus affects the epidermal growth factor (EGF) precursor homology domain of the LDLR protein, encoded by exons 7–14. On the other hand, the positively charged lysine residue found in the detected *LDLR* variant might be affecting the recycling of the receptor (Tai et al., 2001). The p.(Arg3500Trp) *APOB* variant detected in this patient was previously identified in an Asian population and was causing an LOF of *APOB*, a phenotype similar to that produced by p.(Arg3500GLn) *APOB* variant (Choong et al., 1997; Tai et al., 2001). The double *LDLR/APOB* heterozygotes with exaggerated hypercholesterolemia phenotype were still responsive to lipid-lowering treatments. In the same year, another double-heterozygous *LDLR/APOB* carrier was detected and diagnosed with a mild FH phenotype. Even though the double-heterozygous patient carried two variants, his LDL-C levels were lower than that of patients with double mutations in the same *APOB*. Of great interest, the LDL-C level in the patient carrying the p.(G-2) *LDLR* variant, which is located in exon 1, and even in combination with in the (B3500) *APOB* locus, was normalized after being treated with a low dose of statin. This reduction in LDL-C was similar to that of two cases reported previously (Benlian et al., 1996; Tai et al., 2001).

Moreover, Taylor et al. (2010) reported a 15-year-old female carrying p.(Leu479Pro) *LDLR* and p.(Arg3527Gln) *APOB* mutations. The paternal and maternal *LDLR/APOB* variants, respectively, were confirmed by cascade testing (Umans-Eckenhausen et al., 2001; Hadfield et al., 2009). Consequently, the combined effect of both *LDLR/APOB* mutations resulted in a phenotype that is more severe than that of each mutation alone and of homozygous FDB. However, the severity of p.(Leu479Pro) *LDLR* and that of p.(Arg3527Gln) *APOB* variants were less than that of HoFH. The presence of two mutations was only identified with genetic screening and was not clinically assumed given the notable response of the case to statins (Taylor et al., 2010). Bertolini et al. (2013) also detected two related double-heterozygous FH cases carrying the p.(Y419^∗^) *LDLR* and p.(R3558C) *APOB* mutants. Unexpectedly, the LDL-C level of these cases was similar to that observed in the p.(Y419^∗^) *LDLR* heterozygotes (Bertolini et al., 2013). More recently, a wide genetic screening study carried out on affected family members of patients with ADH identified 23 double-heterozygous carriers of the *LDLR/APOB* variants in a cohort of 17 unrelated families from the Netherlands. The levels of LDL-C before treatment were higher in these patients than those of the heterozygotes or the unaffected relatives (Sjouke et al., 2016b). The LDL-C level, however, was significantly lower than that of those patients with homozygous and compound heterozygous *LDLR* variants. Significantly, CVD was present in only 36% of the double-heterozygous *LDLR/APOB* carriers, a value similar to that of homozygous and compound heterozygous ADH (Sjouke et al., 2016b). Although *APOB* mutations are an infrequent cause of FH in Italy, five double-heterozygous carrying the pathogenic *LDLR/APOB* variants were detected in an Italian cohort of 1,076 individuals (Pirillo et al., 2017). The phenotypes of double-heterozygote patients and their response to LLT were not reported by the study. Recently, the sequencing of a cohort of 1,191 patients with FH identified six double-heterozygous patients carrying *APOB/LDLR* mutations. Three of six patients were found to be *APOB*-defective/*LDLR*-negative, and the other three patients were *APOB*-defective/*LDLR-*defective (Hartgers et al., 2018). All patients had a high CVD risk at baseline. The main objectives of the work were to study the efficacy of alirocumab, a PCSK9 antibody, on patients with double-heterozygous FH. For this reason, four patients received alirocumab treatment in addition to a concomitant statin, and two were placebo-treated. The inhibition of PCSK9 with the monoclonal antibody alirocumab reduces LDL-C levels by increasing the level of LDLRs on the liver cell surface, resulting in an increased uptake of the LDL particles. Upon alirocumab treatment, the LDL-C levels dropped down in patients with *APOB-*defective/*LDLR*-negative and *APOB-*defective/*LDLR-*defective at weeks 12 and 24, respectively. These results reveal that double-heterozygous *APOB/LDLR* mutations appeared not to influence the efficacy of alirocumab (Hartgers et al., 2018). Although statins are the first choice for treating FH, PCSK9 inhibitors (including alirocumab) are recently showing more significant LDL-C and atherosclerosis, reducing actions in FH patients.

Usually, the presence of both *LDLR* and *APOB* mutations should be considered in two cases. First, when there is an *LDLR* mutation in some but not all members of a family who exhibit FH, and second, in individuals who have the same *LDLR* mutation but show an exaggerated phenotype compared with other members of the family (Taylor et al., 2010). The severity of the FH phenotype, in addition to CVD risk, may be associated with the combined effect of two pathogenic variations in the two primary candidate genes, *LDLR* and *APOB*. The few studies on the double-heterozygous FH cases carrying both *LDLR* and *APOB* variants reveal that the clinical phenotype is more severe when compared to the simple heterozygous FDB and ADH forms. However, it appears that the phenotype is intermediate between that observed in heterozygous and homozygous FH (Rubinsztein et al., 1993; Benlian et al., 1996; Tai et al., 2001).

### *APOB*/*PSCK9* Mutations

The *PCSK9* gene encodes an enzyme that binds to the epidermal growth factor-like repeat A (EGF-A) to promote the degradation of the LDLR in the lysosome of the cell (Sharifi et al., 2017). *PCSK9* has been heavily investigated in many FH populations where a list of more than 161 variants has been identified along with the 12 exons of the gene. The severity of the phenotype is associated with the type of mutation. *PCSK9* LOF mutations are more likely to be associated with a decrease in the cholesterol levels, whereas GOF mutations can lead to a more severe FH associated with a decrease of LDLR on the surface of the cells. Both *in vitro* and *in vivo* studies have significantly aided in unraveling the pathophysiological function of the *PCSK9* gene in human biology in recent years (Lambert et al., 2012). Since the subsequent discovery of the GOF *PCSK9* mutations that result in FH, many research studies have been published about the clinical features and genotypes of patients with ADH. Many *PCSK9* single-nucleotide polymorphisms (SNPs) have also been discovered that have a differential effect on cholesterol regulation in different populations (Fahed and Nemer, 2011). As *PCSK9* promotes LDLR degradation and prevents its recycling to the membrane, studies are now focusing on PCSK9 inhibitors as an emerging safe therapy for dyslipidemia supported by the fact that LOF mutations in *PCSK9* are associated with reduced LDL-C levels and lower CHD risk (Cohen et al., 2006; Benn et al., 2010; Lambert et al., 2012). So far, very few studies have reported digenic mutations in *PCSK9* and other FH genes such *as LDLR* and *APOB*.

The first and only reported *APOB/PCSK9* case was identified in a patient with FH patient from a French cohort with a novel digenic p.(Ala3396Thr) *APOB*/p.(Arg96Cys) *PCSK9* mutations. The patient presented with severe ADH, including an arcus cornea despite treatment with 10 mg of rosuvastatin and ezetimibe. Despite treatment, the detected *APOB/PCSK9* variants resulted in an elevation of total cholesterol (TC) (201 mg/dL; 5,200 nmol/mL) and LDL-C (130 mg/dL; 3,400 nmol/mL) levels (Elbitar et al., 2018). p.(Ala3396Thr). *APOB* had no phenocopy and was shown to have complete penetrance. *In silico* analysis showed that alanine (Ala) at position 3396 in apoB is highly conserved among different species and could be disease-causing. Exome sequencing showed that no one of the other family members carried the *PCSK9* variant. In this study, *in silico* and functional analysis studies were performed to detect the pathogenicity of the detected variants. The *APOB* variant was confirmed to be pathogenic as it affected the apoB binding site to LDLR, suggesting the disruption of apoB–LDLR interaction and LDL internalization (Elbitar et al., 2018).

Also, the identified *PCSK9* variant revealed a 60% increase in the PCSK9 cellular level *in vitro* when compared with the wild-type and to the well-characterized p.(Ser127Arg) GOF variant. This indicates that the novel identified p.(Arg96Cys) is an active GOF mutant as its overexpression results in higher LDLR degradation than the wild-type, causing LDL-C accumulation. Yet, the p.(Arg96Cys) can alone cause ADH as previously reported while aggravating the phenotype when being expressed with another mutant causing ADH (Hopkins et al., 2015; Elbitar et al., 2018).

As studying the effect of the mutations on protein–protein interactions (PPIs) is still lacking in most of the reported studies, we believe that investigating PPIs can be fundamental in understanding the phenotypic changes as the functional interactions are created or disrupted. As other FH cases carrying both *APOB/PCSK9* variants might exist, the molecular identification of FH double-heterozygotes is still essential through genetic cascade screening to provide proper diagnosis and treatment for FH.

### LDLR/PCSK9 Mutations

*PCSK9* has been identified as an FH modifier gene as it generates significant variable phenotypes even in patients having the same mutation in LDLR. Nowadays, many studies have put looking for *PCSK9* mutations on the top of *LDLR* variants (Abifadel et al., 2009). Mutations in the LDLR protein are distributed through the domains and can lead to various types of dysfunctions. For instance, null mutations generate no LDLRs. In other cases, defects in LDLR can affect its localization to the nuclear membrane, internalization into the cell after binding to LDL-C, or binding to LDL-C particle (Brown and Goldstein, 1986).

It is well known now that homozygous or compound heterozygous FH patients with *LDLR* mutations or double-heterozygotes carrying *LDLR* and p.(R3500Q) *APOB* mutations have more severe phenotypes when compared to the heterozygote FH cases carrying only one mutation in any of the mentioned genes or for missense mutations in the *PCSK9* gene. It was not very clear whether double-heterozygous FH cases carrying *LDLR*/*PCSK9* mutations can lead to similar severe phenotype until Pisciotta et al. (2006a) reported two unrelated double-heterozygous FH carrying *LDLR/PCSK9* mutations with clinical phenotype and family history similar to that of the HoFH. One of the patients carried the two novel p.(N425S) *PCSK9* and p.(Y419X) *LDLR* mutations. The other patient was a carrier of a previously reported variant p.(E228K) *LDLR* variant and a novel p.(R496W) *PCSK9* variant. The cases presented in this study revealed more severe phenotypes as their LDL-C levels were 56 and 44% higher than those relatives who were just carriers of the LDLR heterozygous mutation. However, the *LDLR/PCSK9* double-heterozygotes displayed premature CAD, xanthomatosis, and carotid atherosclerosis (Pisciotta et al., 2006a). As previously reported, the p.(E228K) *LDLR* variant was speculated to produce a severe defective *LDLR* with <2% residual receptor activity, which was similar to the severity of the phenotype of this patient (Hobbs et al., 1992). The novel p.(R496W) *PCSK9* variant, which was also present in the patient’s HeFH mother (Bertolini et al., 2004), suggested the high pathogenicity of the *PCSK9* variant might be involved in decreasing the LDLR activity. As the *PCSK9* missense mutations affect the highly conserved amino acids of the proteins, it is suggested that those mutations might be affecting the functional properties of the PCSK9 protein. This implies that a missense mutation in *PCSK9* along with another *LDLR* mutation might lead to a more severe clinical FH phenotype (Taylor et al., 2010; Pisciotta et al., 2006a). Unpredictably, the *LDLR/PCSK9* double-heterozygotes responded to LLT and obtained up to 70% reduction in LDL-C, a level that is rarely attained with the HoFH patients (Pisciotta et al., 2006a). Moreover, in another study with a total of 28 double-heterozygotes, five cases were identified as carriers of *LDLR/PCSK9* mutations. Three patients carried the c.(191-2A. G) *LDLR*/p.(Arg476Cys) PCSK9 variants, and two carried the p.(Val429Met) *LDLR*/(p. Ala53Gly) *PCSK9* variant*s*. Although *LDLR/PCSK9* individuals had higher LDL-C levels than heterozygotes and unaffected individuals, their TC and LDL-C levels were similar to the carriers of homozygous or compound heterozygous *LDLR* mutations (Sjouke et al., 2016b). However, this came in contrast with a previous study that reported the LDL-C levels of *LDLR/PCSK9* double-heterozygous carriers were less severe than those of HoFH (Cuchel et al., 2014).

Another severe double-heterozygous *LDLR/PCSK9* FH case was also recently reported. The patient was identified with p.(Cys143) *LDLR* and p.(Leu22_Leu23dup) *PCSK9* variants. After 12 weeks of treatment with 150 mg alirocumab, a PCSK9 inhibitor, the patient was highly responsive to the treatment (Hartgers et al., 2018). The inhibition of PCSK9 by alirocumab (monoclonal antibodies) reduces the LDL-C level through increasing the level of LDLR on hepatocytes. In support of previous studies, the results obtained suggest that *PCSK9* GOF variants do not impair alirocumab efficacy (Hopkins et al., 2015; Hartgers et al., 2018). In another recent study, a double-heterozygous *LDLR*/p.(V4I) *PCSK9* FH patient received a PCSK9 antibody associated with coronary plaque regression due to coronary artery syndrome. In addition to CAD, the clinical manifestations included very high levels of LDL-C and extensive xanthomas. The combination of an *LDLR* mutation with a p.(V4I) *PCSK9* mutation in clinically diagnosed FH yields a severe phenotype and makes the patient to be more prone to CAD (Ohta et al., 2016; Shirahama et al., 2018). Lipid-lowering drugs, including rosuvastatin (20 mg) and ezetimibe (10 mg), were not sufficient to achieve a reduction as per the guidelines. Therefore, evolocumab (monoclonal PCSK9 antibody) was also introduced and reduced the LDL-C level (Shirahama et al., 2018).

As PCSK9 inhibits LDLR recycling, which decreases the number of LDLR on the cell surface, inhibiting PCSK9 protein can increase the number of available LDLR protein on the cell surface and increase in return the uptake of LDL-C into the cell (Handelsman and Lepor, 2018). Numerous studies conducted on cells, humans, and animals showed that LOF *PCSK9* mutations could elevate the level of hepatic *LDLR* and thus decrease the amount of LDL-C in circulation (Seidah, 2017). *PCSK9* knockout (KO) mice revealed a hypocholesterolemia phenotype, yielding an 80% decrease in LDL-C. Besides, the KO mice displayed a better response to statins and had a reduced risk for atherosclerosis (Rashid et al., 2005; Zaid et al., 2008; Denis et al., 2012). However, an opposite phenotype was observed in transgenic mice and pigs overexpressing the wild-type *PCSK9* or its GOF mutant p.(D347Y) (Denis et al., 2012; Al-Mashhadi et al., 2013, 2015). Therefore, the promising outcomes of novel therapeutic approaches using PCSK9 inhibitors can improve the severity of the FH phenotype through lowering LDL-C, thus preventing the progression of CAD.

### *LDLR*/*LDLRAP* Mutations

The *LDLRAP1* gene, located on the short arm of chromosome 1, encodes the cytoplasmic LDLRAP1 protein that acts as an adaptor for LDLR endocytosis in hepatic cells (Sharifi et al., 2017). Usually, mutations in this gene cause premature truncations of the protein, thus leading to the disruption of LDLR. In these cases, the LDL-C level is commonly intermediate between homozygote and heterozygote ADH patients (Austin et al., 2004; Michaely et al., 2004). Mainly, null mutations in *LDLRAP1* can lead to ARH (Garcia et al., 2001). There are some phenotypic differences between ARH and FH-causing *LDLR* mutations, where ARH is typically less severe and more responsive to LLT (Soutar and Naoumova, 2007). However, there is tremendous phenotypic variability among the ARH patients in general, and ARH patients of the same family (Pisciotta et al., 2006b). As ARH is usually uncommon, there are few published data on heterozygous carriers of *LDLRAP1* variants. Data on the clinical manifestation of having *LDLR*/*LDRAP1* double-heterozygotes are also rare. Until now, only four studies have reported cases with digenic mutations in *LDLR* and *LDLRAP1* genes.

The first case of *LDLR/LDLRAP1* was reported in a cohort of 146 individuals where three Japanese FH patients of the same family were double-heterozygous for a nonsense *LDLR* p.(Lys790^∗^) variant and a frameshift p.(Lys204-Glufs^∗^17) *LDLRAP1* variant. The latter was previously reported by the same group to cause elevated LDL-C levels when compared to individuals not carrying this mutant. Only one patient out of three had severe FH phenotype reflected by extensive xanthomas and coronary and aortic valvular disease. However, the LDL-C levels in this patient and the other two patients were similar to individuals with single *LDLR* mutations. Remarkably, the family members showed different LDL-C levels, which suggests the presence of another unknown player (Tada et al., 2011). Soufi et al. (2013) identified four other *LDLR/LDLRAP1* double-heterozygotes in a Turkish FH family. The patients carried the p.(Q136^∗^) *LDLRAP1* homozygous variant along with a p.(Q254P) *LDLR* heterozygous variant. The double-heterozygotes needed weekly apheresis as they did not reach the target LDL-C level by lipid-lowering drugs. The severity of phenotype in the double-heterozygotes was manifested by the presence of xanthomas and CVD. In previous studies, the detected mutation in *LDLR* has shown to affect the ligand-binding domain, while the *LDLRAP1* mutant was generating a truncated protein with a missing phosphotyrosine-binding domain that is needed for LDLR internalization (Garcia et al., 2001; Guardamagna et al., 2009). The authors thus concluded that the combination of the heterozygous *LDLR* and the homozygous *LDLRAP1* variants leads to a more severe phenotype compared to that of homozygous *LDLR* variant alone (Soufi et al., 2013).

Additionally, Fahed et al., 2016 reported Lebanese family members with FH who were double-heterozygotes. The patients carried a p.(Q136^∗^) *LDLRAP1* variant along with homozygous and heterozygous mutations in *LDLR* including p.(C681^∗^), p.(H327fsX5), p.(A391T), and p.(I451T). The prevalence of *LDLRAP1* was unraveled as another founder mutation in the Lebanese population as all the affected family members carried the p.(Q136^∗^) *LDLRAP1* variant. The patients with these mutations, however, revealed variable phenotypic expressivity ranging from mild to severe along with either standard or high LDL-C levels. Although no universal pattern has been identified in this study, the arrangement of the homozygous variant of a gene with a heterozygous variant of another gene accounted for a severe dramatic increase in the levels of LDL-C. Although the occurrence of three heterozygous variants did not similarly increase the levels of LDL-C (Fahed et al., 2016).

Recently, Alnouri et al. (2018) identified four double-heterozygous *LDLR/LDLRAP1* individuals, a mother and three of her children in a Saudi cohort. The reported digenic mutations included the novel p.(Y419D) *LDLR* and p.(S202Tfs^∗^2) *LDLRAP1* variants. The double-heterozygous mother showed a severe FH phenotype (including high LDL-C, xanthomas, and CAD), suggesting that that the additional *LDLRAP1* mutation leads to a more severe phenotype for xanthoma and atherosclerotic CVD in FH patients. Upon using the lipid-lowering drugs, rosuvastatin (40 mg) and ezetimibe (10 mg), the patient’s LDL-C decrease did not reach the optimal level recommended by the European Society of Cardiology. Surprisingly, her children with the same mutations had no clinical manifestations. Severe clinical events obtained in the mother were associated with the effect of the mutations on the functional activity of the protein. Using *in silico* analysis, p.(Y419D) *LDLR* variant was predicted to be deleterious through disrupting the EGF-A domain of the protein. Additionally, the frameshift mutation in *LDLRAP1* generated a truncated protein lacking an essential functional domain that might be affecting the cellular internalization of LDLR/LDL-C (Alnouri et al., 2018).

Although it is not fully understood why the same allele generates subtly or profoundly different phenotypes in most of the FH cases, the outcomes of the studies reporting digenic mutations *LDLR/LDLRAP1*, although very few, can be fundamental for (1) understanding better the phenotype–genotype correlations and (2) further elucidation of the FH heterogeneity and the mutation spectrum.

## Digenic FH: A Spectrum of Phenotypic Diversity

Familial hypercholesterolemia is a heterogeneous disease where patients show a significant variability of phenotypes that may be explained by the polygenic nature of the disease, which has been lately enhanced by the characterization of modifier genes such as *PCSK9*. Typically, the severity of phenotype varies with the genotype (Sun et al., 2018). This variation is reflected by differences in the phenotypes and the clinical severity of untreated HeFH, double HeFH, compound HeFH, and HoFH, with some overlap within and between genotypes. Also, treated HeFH and HoFH might have an overlap in LDL-C levels (Foody and Vishwanath, 2016). Mutations in more than one gene are predicted, in most cases, to worsen the phenotype and severely increase untreated LDL-C levels. However, this was not the case all the time in FH cases with digenic mutations.

In some cases, the severity of the phenotypes of double-heterozygotes was similar to HeFH, yet not as severe as in HoFH (Mabuchi et al., 2014). For example, the range of LDL-C values associated with double-heterozygotes seems to be broader compared with homozygotes from 196 mg/dL (5,100 nmol/mL; *LDLR/PCSK9*) to 583 mg/dL (15,100 nmol/mL; *LDLR/APOB*) (Benlian et al., 1996; Mabuchi et al., 2011). In one study, 13 heterozygous carriers of either *LDLR/APOB* or *LDLR/PCSK9* mutations were identified by NGS in 285 unrelated Chinese index cases of clinical FH (279 adults, six children). Eight patients out of 13 were treated with statins, and four had a CAD family history. Yet, the double-heterozygotes showed a much milder phenotype compared to the compound and true homozygotes (Sun et al., 2018). Occasionally, double-heterozygotes (a combination of *LDLR* and *APOB/PCSK9* mutations) reflected an intermediate phenotype mainly due to the milder phenotypes of *APOB/PCSK9* carriers (Sánchez-Hernández et al., 2016; Sjouke et al., 2016b). However, as the lipid levels detected in double-heterozygotes were relatively low, the authors claim that this contradiction in data presentation might be related to the small sample size (Sun et al., 2018). Although the range of LDL-C levels is more comprehensive, some double-heterozygotes may often have higher amounts than individuals with true HoFH or compound HeFH. For example, the LDL-C levels in double-heterozygotes having the *LDLR*/*PCSK9* mutations would be expected to vary to a greater or lesser amount compared with levels in individuals with the *LDLR* mutation alone. However, this usually depends on whether the *PCSK9* mutation provides a (1) GOF, leading to FH due to the increase in LDLR degradation, or (2) an LOF leading to hypercholesterolemia associated with the decrease in LDLR degradation (Noguchi et al., 2010; Mabuchi et al., 2011). In one of the cases, the clinical features of the FH individuals with double-heterozygous mutations were similar to those of family members with only one heterozygous mutation (Rauh et al., 1991). One explanation for the lack of difference between double-heterozygous (digenic) and the monogenic heterozygous FH cases is that one of the variants involved is not pathogenic and indeed is a synonymous mutation. It is obviously of great importance to be capable of assessing whether the identified variants are pathogenic or not especially that predicting the pathogenicity of novel variants is not always straightforward. The gold standard to test the pathogenicity of any variant was to perform cosegregation studies with the high LDL-C level among family members. However, the interpretation of clinical data could be puzzling as other environmental and genetic factors variants might be involved in raising up or lowering LDL-C levels (Sharifi et al., 2017). This counterintuitive finding highlights the need for better *in silico* tools and more *in vitro* functional studies to define the effect of a mutation on the protein function and structure. In addition, the differences in phenotypes could be also explained by the presence of other genetic events taking place in some other unknown genes, which reflects the importance of revealing the cause of phenocopies when studying familial segregations.

The heterogeneity and variability in clinical phenotypes within the spectrum of FH mutation carriers propose that the detection and diagnosis of double-heterozygous FH are very difficult and can sometimes be missed. It is not yet clear whether the genetic diagnosis of FH patients as double-heterozygotes is clinically relevant, because in most of the cases, the level of LDL-C is sufficiently treated with lipid-lowering drugs, including statins and PCSK9 inhibitors. Still, many patients who are clinically diagnosed with FH do not reveal any mutation in the four FH genes even with genetic testing. This suggests the presence of many unknown genes involved in cholesterol metabolism that needs to be detected to fill the diagnostic gaps of FH (Fahed and Nemer, 2011).

With the understanding of the FH genetic basis, studies have illustrated that the pronounced increase in LDL-C level was by far due to more than the common monogenic FH. Other causes of FH can be referred to (1) the presence of rare mutations in the known FH genes, (2) mutations in a novel gene, and (3) polygenic FH secondary to the cumulative effect of LDL-C raising SNPs, and (4) other acquired phenocopies (Kwon et al., 2015; Sjouke et al., 2016a). Consequently, performing NGS for all affected and non-affected familial members might aid in filling the gap for the establishment of phenotype–genotype correlations in FH (Fahed et al., 2016).

## Promiscuity of Phenotype–Genotype Correlation: NGS and FH

The introduction of NGS technologies during the last decade is leading to an exponential elevation in the discovery and differentiation between variants of variable functional significance. Those could be classified based on several measures as either clearly or likely pathogenic, of unknown significance, or unlikely pathogenic, or clearly not pathogenic. When DI comes into play, things become more complicated as two or more variants are involved in defining and justifying symptoms and diagnosis, respectively (Deltas, 2018). In other words, NGS is a highly reliable tool in elucidating the entire spectrum of variants in individuals, including those that cause digenic or polygenic disorders. Because of its massively parallel sequencing abilities, NGS is currently considered the primary high-throughput diagnostic tool for FH genetic testing, including all aspects of targeted exome sequencing, whole-exome sequencing (WES), and whole-genome sequencing (WGS). Even though genetic testing and family-based cascade screening are considered high-cost approaches for the diagnosis of FH, they are still useful in distinguishing the different forms of FH (Nherera et al., 2011; Nordestgaard et al., 2013; Talmud et al., 2013).

Although FH diagnosis can be built on several clinical manifestations, the Dutch Lipid Clinic Network Diagnostic Criteria has included genetic testing as an essential tool for more precise disease diagnosis (Defesche et al., 2004; Sturm et al., 2018). Meanwhile, the gold standard for FH diagnosis is to identify a pathogenic variant for FH other than those affecting the function of *LDLR* (Humphries et al., 2008; Watts et al., 2014). Thus, the detection of one or more pathogenic mutations in *LDLR*, *APOB*, or *PCSK9* by genetic testing offers a more definite molecular diagnosis of FH. Detecting a pathogenic variant(s) in the FH proband facilitates the family cascade genetic testing in the at-risk relatives. It can provide precise results for with and without FH relatives.

At the molecular level, genetic testing makes it easier to distinguish FH individuals with (1) heterozygous, (2) double heterozygous, (3) compound heterozygous, (4) homozygous, (5) autosomal recessive mutations, and (6) patients with no detectable pathogenic variant and yet have FH phenotype. The risks of recurrence to relatives and implications for family planning vary between these states. For example, in scenarios where the results of genetic tests recognize double-heterozygotes probands (for example, pathogenic mutations in both *LDLR* and *APOB*), the recurrence risk to relatives is affected by this finding and the recommended approaches to cascade testing (Sturm et al., 2018). As for probands whose genetic testing specifically identifies them as a compound, double-heterozygotes, or homozygotes, parents of the probands should undergo known familial variant testing to identify (1) the maternally and paternally inherited variants, (2) determine whether one of the variants is *de novo*, and (3) which is rare (Tada et al., 2016).

No doubt, NGS technologies have shown to be essential tools for identifying new mutations in FH genes and in revealing double-heterozygous mutations as it improves familial screening, genetic counseling, and understanding disease severity and transmission in different members of the same family (Elbitar et al., 2018). For example, huge genomic testing using WES done on a cohort of 50,726 individuals in a healthcare center in the United States revealed the that frequencies of mutations in *LDLR*, *APOB*, and *PCSK9* were 42.8, 44.5, and 12.7%, respectively (Abul-Husn et al., 2016). Similarly, using WES, Wang et al. (2016) reported 47.3% definite or likely pathogenic variants out of the 313 Ontario-based hypercholesterolemia cohort. Of 105 detected mutations, 87.5% were located with *LDLR* and only 12.5% in *APOB* and *PCSK9*. Interestingly, the study also reported 16 novel mutations, 12 of which in *LDLR* gene, with five missense, five frameshift, and two splicing mutations (Wang et al., 2016). Khera et al. (2019) performed WGS on a 2,081 United States–based cohort. Irrespective of their lipid status, the cohort was recruited after the early onset of myocardial infarction (MI). Sequencing analysis indicated 1.7% of the cases have FH-causing mutations in *LDLR* and surprisingly with no mutations detected in neither *APOB* nor *PCSK9* (Khera et al., 2019). Additionally, WGS data retrieved from 2,081 patients with early-onset MI showed that 17.1% of individuals with a high polygenic score had no mutation in *LDLR*, *APOB*, or *PCSK9*. In contrast, only 0.2% had both high polygenic score and a variation in the FH primary genes (Khera et al., 2019). Genetic cascade testing is recommended by United Kingdom guidelines to identify affected relatives; however, approximately 60% of patients are mutation-negative (Talmud et al., 2013).

Next-generation sequencing has the upper hand in FH diagnosis, and the drop in the cost of NGS made genetic testing more accessible. However, there is a high need for a significant data interpretation. Although a large number of genetic studies have spotted a broad spectrum of mutations in multiple genes to be associated with high LDL-C and FH, the underlying mechanism on how these mutations are linked to elevated LDL-C level might provide better insight on drug/gene or protein targets.

## Conclusion

As more genes and loci have been additionally identified for monogenic FH, the characterization of new variations in these known genes and loci would assist in the elucidation of novel double-heterozygous FH cases. The polygenic aspect of FH should also be considered, given the fact that some FH phenotypes are due to undescribed variants located in other unknown genomic regions. Although FH is an inherited disorder, genetic testing for the diagnosis of this disease is still infrequently ordered. The genetic testing aids in improving the diagnosis and prognosis of FH, which are hard to be achieved because of the heterogeneity and the variable expressivity of the available mutations and phenotypes. Therefore, additional research is needed to evaluate how data generated from genetic testing can improve medication and outcomes for FH patients. More likely, and as it was the case with PCSK9 drug targets, this might help in introducing novel lipid-lowering therapeutics needed to treat high-risk patients and families.

## Author Contributions

AKa compiled all the published data and wrote the first draft of the manuscript. All authors contributed to the critical review of the data, correction, and final write-up of the manuscript.

## Conflict of Interest

The authors declare that the research was conducted in the absence of any commercial or financial relationships that could be construed as a potential conflict of interest.

## Footnotes

1 https://databases.lovd.nl/shared/genes/LDLR
